# The Immunomodulatory Signature of Extracellular Vesicles From Cardiosphere-Derived Cells: A Proteomic and miRNA Profiling

**DOI:** 10.3389/fcell.2020.00321

**Published:** 2020-06-09

**Authors:** Esther López, Federica Marinaro, María de los Ángeles de Pedro, Francisco Miguel Sánchez-Margallo, María Gómez-Serrano, Viviane Ponath, Elke Pogge von Strandmann, Inmaculada Jorge, Jesús Vázquez, Luis Miguel Fernández-Pereira, Verónica Crisóstomo, Verónica Álvarez, Javier G. Casado

**Affiliations:** ^1^Stem Cell Therapy Unit, Jesús Usón Minimally Invasive Surgery Centre, Cáceres, Spain; ^2^CIBER de Enfermedades Cardiovasculares (CIBERCV), Madrid, Spain; ^3^Laboratory of Cardiovascular Proteomics, Centro Nacional de Investigaciones Cardiovasculares (CNIC), Madrid, Spain; ^4^Institute of Molecular Biology and Tumor Research (IMT), Center for Tumor Biology and Immunology (ZTI), Philipps University, Marburg, Germany; ^5^Institute for Tumor Immunology, Center for Tumor Biology and Immunology (ZTI), Philipps University, Marburg, Germany; ^6^Clinic for Hematology, Oncology, and Immunology, Philipps University, Marburg, Germany; ^7^Immunology Department, Hospital San Pedro de Alcántara, Cáceres, Spain

**Keywords:** cardiosphere-derived cells, cardiac stem cells, proteomic analyses, quantitative polymerase chain reaction, interferon-γ, extracellular vesicles, miRNA–microRNA, priming

## Abstract

Experimental data demonstrated that the regenerative potential and immunomodulatory capacity of cardiosphere-derived cells (CDCs) is mediated by paracrine mechanisms. In this process, extracellular vesicles derived from CDCs (EV-CDCs) are key mediators of their therapeutic effect. Considering the future applicability of these vesicles in human diseases, an accurate preclinical-to-clinical translation is needed, as well as an exhaustive molecular characterization of animal-derived therapeutic products. Based on that, the main goal of this study was to perform a comprehensive characterization of proteins and miRNAs in extracellular vesicles from porcine CDCs as a clinically relevant animal model. The analysis was performed by identification and quantification of proteins and miRNA expression profiles. Our results revealed the presence of clusters of immune-related and cardiac-related molecular biomarkers in EV-CDCs. Additionally, considering that priming stem cells with inflammatory stimuli may increase the therapeutic potential of released vesicles, here we studied the dynamic changes that occur in the extracellular vesicles from IFNγ-primed CDCs. These analyses detected statistically significant changes in several miRNAs and proteins. Notably, the increase in interleukin 6 (IL6) protein, as well as the increase in mir-125b (that targets IL6 receptor) was especially relevant. These results suggest a potential involvement of EV-CDCs in the regulation of the IL6/IL6R axis, with implications in inflammatory-mediated diseases.

## Introduction

Cardiac-derived stem cells have been considered as one of the most promising therapeutic options for myocardial regeneration (Zhang et al., 2015; Lader et al., 2017). However, more than 30 top-cited articles have been retracted in the last year (Chien et al., 2019). In the early years of stem cell-based therapies, several disappointing results were reported after the administration of MSCs in myocardial infarction (Miao et al., 2017). Some years later, clinical trials were focused on the administration of cardiac stem cells, and 5 years ago, the clinical trial CADUCEUS (ClinicalTrials.gov Identifier: NCT00893360) opened an optimistic scenario in cardiology, demonstrating the regenerative potential of autologous CDCs (Malliaras et al., 2014).

Nowadays, accumulating pieces of evidence have demonstrated that paracrine mechanisms have a major impact on immunomodulation and tissue regeneration capacity of stem cells (Epstein, 2018). In this sense, exosomes derived from CDCs have demonstrated a therapeutic effect (Ibrahim et al., 2014). This was further confirmed in a clinically relevant animal model of acute and chronic myocardial infarction, where exosomes also demonstrated a relevant clinical outcome (Gallet et al., 2016).

Considering these results, different groups have tried to unravel the molecular mechanisms underlying the therapeutic effects of CDCs and their EVs. In this sense, *in vitro* and *in vivo* studies in murine models using EV-CDCs and their most abundant small RNA constituent, the Y RNA fragment YF1, produced an increase in the anti-inflammatory cytokine IL10 levels, inducing cardioprotection and attenuating hypertension-associated damage (Cambier et al., 2017, 2018).

*In vivo* studies in rats and pigs have also demonstrated that exosomes from CDCs reduce the presence of infiltrating macrophages in the infarcted tissue and mediate macrophage polarization through miRNAs, such as mir-181b (de Couto et al., 2017). Furthermore, the analysis of miRNAs in exosomes from CDCs cultured under hypoxic conditions increased pro-angiogenic miRNAs (mir-126, mir-130a, and mir-210) (Namazi et al., 2018a) as well as helped in the release of exosomes with anti-apoptotic properties (Namazi et al., 2018b).

Taking together the therapeutic effect of CDC-derived EVs and their promising application in different diseases, such as Duchenne muscular dystrophy (Aminzadeh et al., 2018), the first goal of this study was to identify biomarkers, or clusters of biomarkers, that might be associated with the therapeutic efficacy of EV-CDCs. A detailed characterization and classification of the proteome was performed by high-throughput proteomic screening, followed by bioinformatic analyses.

Furthermore, an innovative aspect of our study lies in the characterization of EVs isolated from IFNγ-primed CDCs (IFNγ/EV-CDCs). The idea of priming adult stem cells with IFNγ to increase their immunomodulatory or pro-regenerative effect is not new, and this effect has been experimentally demonstrated in MSCs from umbilical cord blood (Oh et al., 2008) and human adipose tissue (DelaRosa et al., 2009). More recently, several studies have been focused on *in vitro* stimulation protocols to trigger the release of vesicles loaded with therapeutic agents. In this regard, primed MSCs (exposed to hypoxia and serum deprivation) released exosomes with increase in the immunomodulatory potential (Showalter et al., 2019), inflammation-primed MSCs amplified EVs’ immunosuppression against T-cell proliferation (Di Trapani et al., 2016), and interleukin-1β-primed MSCs produced exosomes with an increased expression of mir-146a with immunomodulatory properties (Song et al., 2017). It is important to note that the inflammatory priming of MSCs has been recently used for donor selection using miRNAs as biomarkers (Ragni et al., 2019).

Apart from protein characterization and considering that miRNA cargo has a key role in the effector function of EVs (Qiu et al., 2018), this study has been also focused on the characterization of a large panel of miRNAs. These miRNAs were selected for their involvement in cardiac regeneration, immune response, and expression in EVs derived from adult stem cells.

To our knowledge, this is the first study describing the proteomic and miRNA profiling of IFNγ/EV-CDCs from a clinically relevant animal model. Here, we show the identification, quantification, and classification of proteins according to immune-related and cardiac-related categories. The presence of interleukin 6 (IL6) in the proteomic analysis is especially relevant, as well as the expression of different miRNAs targeting interleukin 6 receptor (IL6R). Altogether, these results highlight a critical role for IL6/IL6R axis in the therapeutic effect of EV-CDCs.

## Materials and Methods

### Isolation and Characterization of CDCs

CDCs were isolated from cardiac explants of four euthanized healthy large white pigs. This procedure was authorized by the Animal Welfare and Ethics Committee of the Jesús Usón Minimally Invasive Surgery Centre, in accordance with the recommendations outlined by the local government (Junta de Extremadura), and the EU Directive 2010/63/EU of the European Parliament on the protection of animals used for scientific purposes.

Briefly, explants were mechanically disaggregated and subjected to three successive enzymatic digestions with a solution of 0.2% trypsin (Lonza, Basel, Switzerland) and 0.2% collagenase IV (Sigma, St. Louis, MO, United States). Cell culture, isolation, and *in vitro* expansion were performed as previously described by our group (Blázquez et al., 2016).

### IFNγ Treatment, Isolation, and Characterization of EV-CDCs

EV-CDCs were isolated from expanded CDCs at passages 12–15 and 80% confluence. For preconditioning, cells were treated with 3 ng/ml swine IFN gamma Recombinant Protein (IFNγ, catalog number RP0126S-025; Kingfisher Biotech, Saint Paul, MN, United States) for 3 days in standard culture medium. Controls and preconditioned cells were washed with PBS and incubated with DMEM containing 1% insulin–transferrin–selenium (product code: 41400045; Thermo Fisher Scientific, Waltham, MA, United States). This conditioned medium was collected at day 4 and centrifuged first at 1,000 × *g* for 10 min at 4°C, and then 5,000 × *g* for 20 min at 4°C. Supernatants were filtered through a 0.22-μm mesh to eliminate dead cells and debris. The filtrate was used to concentrate the EV-CDCs through a 3-kDa MWCO Amicon^®^ Ultra device (Merck-Millipore, MA, United States) by centrifugation at 4,000 × *g* for 1 h at 4°C. Concentrate samples were recovered from the device and stored at −20°C until further analyses.

The characterization of EV-CDCs was performed by high-throughput proteomic analysis, and proteins were classified following the MISEV2018 guidelines of the International Society for Extracellular Vesicles (ISEV) (Théry et al., 2018). In compliance with ISEV recommendations, EV preparations should be characterized by at least three positive protein markers. Accordingly, a total of 88 proteins from our EV-CDCs were grouped in the following categories: “Transmembrane or GPI-anchored proteins associated to plasma membrane and/or endosomes”, “Cytosolic proteins recovered in EVs”, “Major components of non-EV co-isolated structures”, “Transmembrane, lipid-bound, and soluble proteins associated to other intracellular compartments than PM/endosomes”, and “Secreted proteins recovered with EVs”. Supplementary Table S1 shows the classification of proteins according to these categories.

In addition to proteomics, a Flow NanoAnalyzer (NanoFCM INC., United Kingdom) was used for the multiparameter analysis of EV-CDCs samples. The Flow NanoAnalyzer platform enables quantitative and multiparameter analysis of single EVs down to 40 nm, which is distinctively sensitive and high throughput. All experiments were performed in compliance with the NanoFCM system’s recommendations (more information on http://www.nanofcm.com/). Briefly, dilution of all samples was individually tested in order to record a total number of events in between 3,000 and 12,000. Concentrated DMEM 1% insulin–transferrin–selenium medium was used for threshold setting and as a blank. Monodisperse silica nanoparticles cocktail (68–155 nm. Cat. No. S16M-Exo; NanoFCM INC.) were employed as the reference to calibrate the size of EVs and polystyrene 210 nm beads (QC Beads; Cat. No. S08210; NanoFCM INC.) at 1:100 dilution for particle concentration estimation. Light scattering was used for the measurement of nanoparticle size and size distributions. The EV size range was set at 40-200 nm. All samples were measured with at least two technical replicates.

### Protein Identification by High-Resolution Liquid Chromatography Coupled to Mass Spectrometry

Protein characterization of EV-CDCs and their comparison with IFNγ/EV-CDCs was performed by a high-throughput multiplexed quantitative proteomic approach according to previously described protocols (Jorge et al., 2009; Navarro and Vázquez, 2009; Bonzon-Kulichenko et al., 2011; Navarro et al., 2014; García-Marqués et al., 2016). Protein extracts were incubated with trypsin using the Filter Aided Sample Preparation (FASP) digestion kit (Expedeon, San Diego, CA, United States), as previously described (Wiśniewski et al., 2011). The resulting peptides were labeled using 8plex-iTRAQ reagents, according to the manufacturer’s instructions, and desalted on OASIS HLB extraction cartridges (Waters Corporation, Milford, MA, United States). Half of the tagged peptides were directly analyzed by liquid chromatography tandem mass spectrometry (LC-MS/MS) in different acquisition runs, and the remaining peptides were separated into three fractions using the high pH reversed-phase peptide fractionation kit (Thermo Fisher Scientific). Samples were analyzed using an Easy nLC 1000 nano-HPLC coupled to a Q Exactive mass spectrometer (Thermo Fisher Scientific). Peptides were injected onto a C18 reversed-phase nano-column (75 μm I.D. and 50 cm; Acclaim PepMap100 from Thermo Fisher Scientific) in buffer A [0.1% formic acid (v/v)] and eluted with a 300-min lineal gradient of buffer B [90% acetonitrile, 0.1% formic acid (v/v)], at 200 nl/min. Mass spectrometry (MS) runs consisted of 140,000 enhanced FT-resolution spectra in the 390 to 1,500-m/z wide range and separated 390–700 m/z (range 1), 650–900 m/z (range 2), and 850–1500 m/z (range 3) followed by data-dependent MS/MS spectra of the 15 most intense parent ions acquired along the chromatographic run. HCD fragmentation was performed at 30% of normalized collision energy. A total of 14 MS data sets, eight from unfractionated material and six from the corresponding fractions, were registered with 80 h total acquisition time.

### Peptide Identification, Protein Quantification, and Statistical Analysis

For peptide identification, MS/MS scans were searched as previously described by Binek et al. (2017) using a combined pig and human database (UniProtKB/Swiss-ProtUniProtKB/Swiss-Prot 20147_02 07 Release). *Sus scrofa* gene and protein annotation is not complete; hence, pig proteins were given priority when they shared peptides with human proteins. The Proteome Discoverer 2.1 software (Thermo Fisher) was used for database searching with the following parameters: trypsin digestion with two maximum missed cleavage sites, precursor mass tolerance of 800 ppm, fragment mass tolerance of 0.02 Da. Variable methionine oxidation (+15.994915 Da) and fixed cysteine carbamidomethylation (+57.021 Da), and 8plex-iTRAQ labeling at lysine and N-terminal modification (+304.2054) were chosen.

For peptide identification, the MS/MS spectra were searched using the probability ratio method (Martínez-Bartolomé et al., 2008), and the FDR of peptide identification was calculated based on the search results against a decoy database using the refined method (Navarro and Vázquez, 2009). Peptide and scan counting were performed assuming as positive events those with an FDR equal or lower than 1%.

Quantitative information of 8plex-iTRAQ reporter ions was extracted from MS/MS spectra using an in-house developed program (SanXoT) as already described (Trevisan-Herraz et al., 2019), and protein abundance changes were analyzed using the WSPP statistical model (Navarro et al., 2014).

Briefly, the log_2_-ratio of concentration in the two samples being compared, A and B, determined by spectrum *s* of peptide *p* derived from protein *q* in experiment *e* is expressed as *X*_eqps_ = log_2_(A/B). The log_2_-ratio value associated with each peptide, *X*_eqp_, is then calculated as a weighted average of the spectra used to quantify the peptide, and the value associated with each protein, *X*_eq_, is similarly the weighted average of its peptides. In addition, a grand mean, *X*_e_, is calculated in each experiment as a weighted average of the protein values. In this study, we calculated *X*_e_ by the integration of the four biological replicates, both from control and IFNγ samples, and determined log_2_ - (*X*_e_IFNγ/*X*_e_ control). WSSP was applied in the SBT workflow that detects significant protein abundance by performing the protein to category integration and taking into account the protein outliers within each category (García-Marqués et al., 2016). For that, proteins were previously annotated based on Gene Ontology database (The Gene Ontology Consortium, 2017). The algorithm provides a standardized variable, *Z*_q_, defined as the mean-corrected log_2_(A/B) expressed in units of standard deviation at the protein level. Student *t*-test was used to compare *Z*_q_ values from EV-CDCs and IFNγ/EV-CDCs, and the statistical significance was set at a value of *p* < 0.05. Enrichment analysis of proteins was performed by DAVID functional annotation database^1^ (Huang et al., 2009a, b) and Benjamini–Hochberg FDR was used for multiple test correction (FDR < 0.05). For biological data interpretation, proteins were classified using the Reactome pathway database^2^ (Fabregat et al., 2018). The mass spectrometry proteomics data have been deposited to the ProteomeXchange Consortium via the PRIDE (Perez-Riverol et al., 2019) partner repository with the dataset identifier PXD016434.

Additionally, PCA was performed on proteins with two or more peptides (number of peptides or Np ≥ 2) quantified after iTRAQ proteomic analysis and at 1% FDR. For PCA, Metaboanalyst software version 4.0^3^ (Chong et al., 2018) was used.

To validate the differential expression patterns shown by proteomic analysis, the expression of IL6 in EV-CDCs and IFNγ/EV-CDCs from three pigs was determined using Porcine IL6 DuoSet ELISA kit (R&D SYSTEMS, Minneapolis, MN, United States). EV samples were normalized by total particle concentration measured by NanoFCM system. ELISA protocol was performed following the manufacturer’s instructions. IL6 concentrations between EV-CDCs and IFNγ/EV-CDCs were compared through a paired *t*-test.

### miRNAs Expression in EV-CDCs and Target Interactions

Expression of the selected miRNAs in EV-CDCs was evaluated by real-time quantitative PCR (qPCR). Total RNAs from EV-CDCs were isolated using mirVANA miRNA isolation kit (Applied Biosystems, Foster City, CA, United States), following the manufacturer’s protocol for total RNA extraction. Quality and concentration of total RNAs were evaluated by spectrophotometry. For reverse transcription, 10 ng of total RNA was used to synthesize miRNAs’ cDNA using TaqMan^®^ Advanced miRNA cDNA Synthesis kit (Cat. No. A28007; Thermo-Fisher Scientific Inc., Waltham, MA, United States), according to the manufacturer’s instructions. Five microliters of diluted cDNA (1:100) was then employed as template for qPCR amplification with the TaqMan^TM^ Fast Advanced Master Mix (Cat. No. 4444964; Thermo-Fisher Scientific Inc., Waltham, MA, United States). Commercial TaqMan^®^ Gene Expression Assays probes (Thermo-Fisher Scientific Inc., Waltham, MA, United States) were used, according to the manufacturer’s recommendations, to evaluate the relative expression of 44 miRNAs (Supplementary Table S2). qPCR reactions were performed in duplicate, and molecular biology-grade water replaced cDNA in no template control reactions. Data from individual TaqMan Assays were acquired by QuantStudio 3 Real-Time PCR System (Applied Biosystems, Thermo Fisher Scientific Inc.) and quantified with the Relative Quantification Application (Thermo Fisher) tool in the Thermo Fisher Cloud software. Levels of each miRNA were normalized to three endogenous controls selected by their score variation. The quantification of miRNAs was performed by 2^–ΔCt^ calculation. Moreover, EV-CDC and IFNγ/EV-CDC differences were compared through paired *t*-test and 2^–ΔΔCt^ calculation (Livak and Schmittgen, 2001).

The miRNet web tool^4^ (Fan and Xia, 2018), that integrates *Sus scrofa* database, was used for miRNA target interaction analysis. Subsequently, Reactome was used to classify the targeted genes according to their biological function.

### *In vitro* Differentiation and Activation of Peripheral Blood Lymphocytes (PBLs), Co-culture With EV-CDCs and IFNγ/EV-CDCs, and Flow Cytometry Analysis

Extracellular vesicles from cardiosphere-derived cells and IFNγ/EV-CDCs were co-cultured *in vitro* with peripheral blood lymphocytes (PBLs) in order to evaluate their immunomodulatory effect. Peripheral blood from one large white pig was collected in EDTA-containing tubes. The blood was diluted in PBS, layered over Histopaque-1077 (Sigma, St. Louis, MO, United States), centrifuged, washed twice with PBS, and seeded in V-bottom 96 well plates at a total density of 200,000 cells per well in RPMI medium. EV-CDCs (*n* = 4) and IFNγ/EV-CDCs (*n* = 4) from four different animals were added to different wells at different concentrations (50, 100, and 200 μg/ml), and analyzed at day 3 by flow cytometry. PBLs without EVs were used as negative control.

For flow cytometry analyses, cells were incubated for 30 min at 4°C with fluorescence-labeled porcine monoclonal antibodies against porcine CD4, CD8α, CD14, CD16, CD27, CD45RA, and Swine Leukocyte Antigen class II (SLAII; AbD Serotec, Kidlington, United Kingdom). Cells were then washed and re-suspended in PBS. Analyses were performed in a FACScalibur cytometer (BD Biosciences, San Jose, CA, United States) after acquisition of 10,000 events. Cells were primarily selected using forward and side scatter characteristics, and fluorescence was analyzed using CellQuest software (BD Biosciences). Appropriate isotype-matched negative control antibodies were used in all the experiments. Paired *t*-test was used to compare each EV dose to the corresponding negative control.

## Results

### High-Throughput Analysis of EV-CDCs and IFNγ/EV-CDCs

**FIGURE 1:**
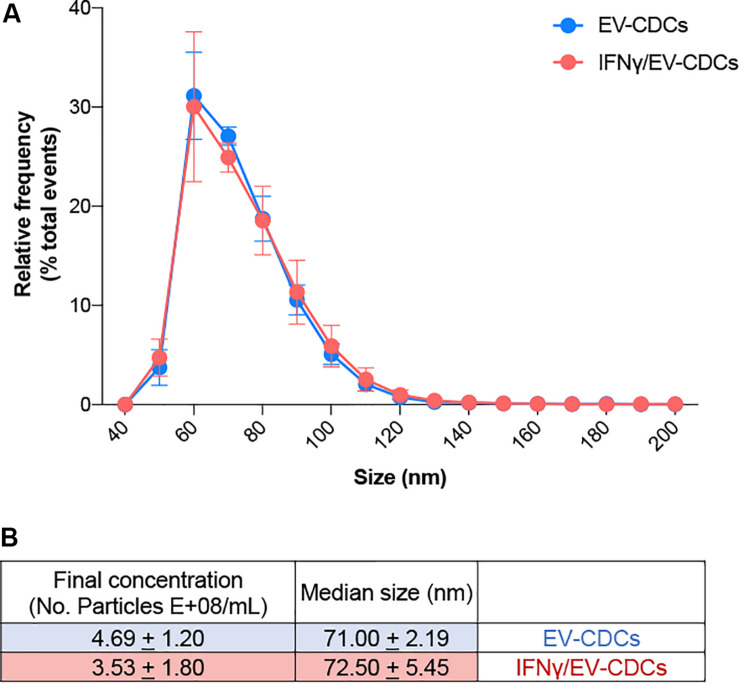
High-sensitivity flow cytometry analysis of extracellular vesicles derived from CDCs (EV-CDCs) at the single-particle level. **(A)** Histogram of particle size with a bin width of 10 nm for control EV-CDCs (blue, *n* = 4) and EVs isolated from IFNγ-primed CDCs (IFNγ/EV-CDCs) (red, *n* = 4) analyzed by NanoFCM. Data show average (±SD) of the relative frequency of total events from representative technical replicates. **(B)** Concentration and median size values of EV-CDC (*n* = 4) and IFNγ/EV-CDC (*n* = 4) samples characterized in this study. Dilution range was tested for every sample, and two technical replicates were recorded for at least 3,000 and up to 12,000 events. Data show the average (±SD) of group samples.

Proteomic profiling provides a global view of subcellular fractions, offering a better understanding of protein abundance. Moreover, quantitative proteomics is a valuable technique for a better characterization of biological products, such as stem cells or stem cell-derived vesicles. In this work, the identification and quantification of EV-CDCs proteins were performed by high-throughput quantitative proteomics using multiplex peptide stable isotope labeling, a useful technique for the characterization of these vesicles (Cypryk et al., 2014). Additionally, high-sensitivity flow cytometry analyses on EV-CDCs samples were performed. Sizing profile of the samples demonstrated that preparations were enriched in small EVs (ranging from 40 to 200 nm) (Figure 1). Median size and concentration of the released EVs showed no significant differences between EV-CDCs and IFNγ-primed CDCs. Besides, nano-FCM analyses performed on non-concentrated conditioned media did not show significant differences in particle releasing between CDCs and IFNγ/CDCs (*data not shown*).

Unfortunately, protein and gene annotation databases for *Sus scrofa* are not as complete as databases for *Homo sapiens.* Thus, MS/MS scans were searched against a combined pig and human database, giving priority to pig identifications when peptide sequences were identified in both (Binek et al., 2017). Quantification of each protein was calculated at the gene-coding level. Of note, 1,205 protein identifications were retrieved only from the pig database. This combined strategy allowed us to increase the depth of the study, depicting around 30% of the identifications (remarkably, 369 identifications were retrieved exclusively from the human database).

**FIGURE 2:**
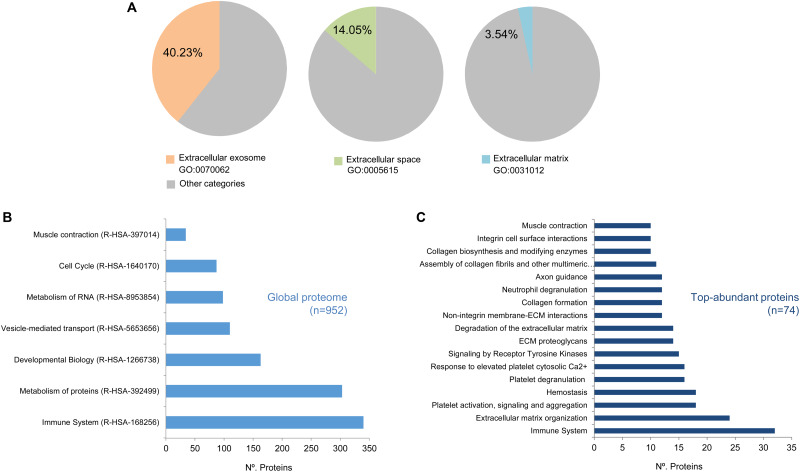
High-throughput proteomic analysis on EV-CDCs. Protein extracts from EV-CDCs (*n* = 4) and IFNγ/EV-CDCs (*n* = 4) were processed by high-resolution liquid chromatography coupled to tandem mass spectrometry analyses. Those proteins were identified by at least two peptides (*n* = 952) and were classified according to their biological function using annotations from the Gene Ontology **(A)** or Reactome **(B,C)** databases. **(A)** Pie charts representing the percentage of proteins (% of total pig annotated proteins) classified as *Extracellular exosome* (GO:0070062, orange), *Extracellular space* (GO:0005615, green), and *Extracellular matrix* (GO:0031012, blue) proteins. Significantly enriched Reactome top-level pathways [enrichment value of *p* < 0.01, 5% false discovery rate (FDR)] including **(B)** the global EV-CDC proteome (*n* = 952, Np ≥ 2) and **(C)** particular most abundant proteins (*n* = 74, Np ≥ 20).

Our study was limited to those proteins represented by at least two peptides (Np ≥ 2). Using this cut-off value, a total of *n* = 952 proteins were analyzed and classified by the DAVID software^5^ (Supplementary Table S3) (Huang et al., 2009a, b). As shown in Figure 2A, this classification revealed that *n* = 375 annotations (40.23% from total annotations) were comprised in the *extracellular exosome* category (GO:0070062), *n* = 131 (14.05%) were comprised in the *extracellular space* category (GO:0005615), and *n* = 33 (3.54%) in the *extracellular matrix* category (GO:0031012). Additionally, the EV-CDCs proteome included 75 proteins from the 100 top-identified proteins in ExoCarta database^6^ (Keerthikumar et al., 2016).

The 952 proteins were then classified according to the Reactome database (Fabregat et al., 2018) to elucidate the functional pathways. Reactome is a hierarchically classified database divided in 24 top-level pathways (such as *Metabolism of Proteins*, *Signal Transduction*, *Immune System*) that serve as “roots” for thousands of more specific pathways. Figure 2B represents the number of proteins classified in the following top-level pathways: *Metabolism of Proteins* (R-HSA-392499), *Signal Transduction* (R-HSA-162582), *Immune System* (R-HSA-168256), *Cell Cycle* (R-HSA-1640170), *Metabolism* (R-HSA-1430728), *Developmental Biology* (R-HSA-1266738), *Metabolism of RNA* (R-HSA-8953854), *Transport of Small Molecules* (R-HSA-382551), *Vesicle-Mediated Transport* (R-HSA-5653656), and *Muscle Contraction* (R-HSA-397014). It is important to note that the number of proteins included in these top-level pathways is directly correlated with the total amount of proteins pre-classified in the Reactome database. Taking into account this observation, an enrichment analysis of the top-abundant proteins (*n* = 74, Np ≥ 20) was performed to identify over-represented pathways (enrichment analyses were calculated using a value (−Log) adjusted by Benjamini–Hochberg FDR correction of *p* ≤ 0.05). Our analysis for the global EV-CDC proteome (*n* = 952, Np >2) demonstrated an enrichment of several top-level pathways: *Immune System*, *Vesicle Transport*, and *Muscle Contraction* (Figure 2B). The identification of more than 300 proteins in the *immune system* pathway was especially relevant. Additionally, the enrichment analysis of top-abundant proteins highlighted several subcategories, such as *Neutrophil Degranulation*, *Platelet Activation*, *Signaling and Aggregation*, and *Degradation of the Extracellular Matrix*, among others (Figure 2C).

**FIGURE 3:**
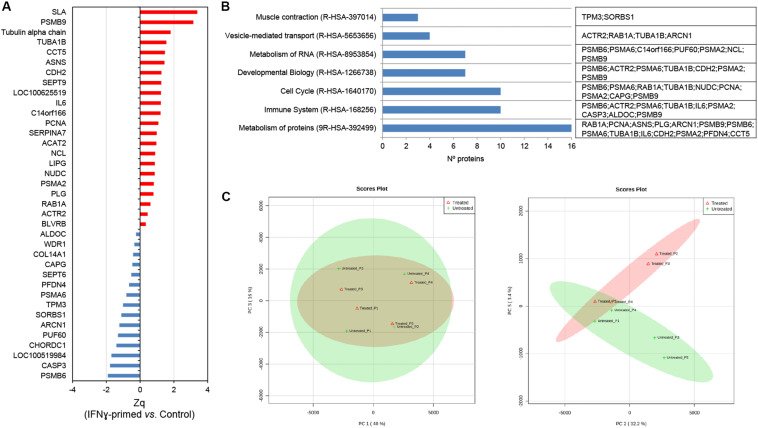
Quantitative proteomic analysis of IFNγ/EV-CDCs. **(A)** Quantitative proteomic results. Protein profile changes were analyzed by the WSPP model (Navarro et al., 2014) to identify significantly altered proteins comparing IFNγ/EV-CDCs and EV-CDCs samples. Since a combined database (human and pig) for peptide searching was used, data were integrated at the gene coding level. Protein (gene) values (Zq) are reported as the standardized variable, which is defined as the mean corrected log_2_-ratio expressed in units of standard deviation. The protein ratio of each sample was calculated against an internal standard (IS) based on the average of iTRAQ reporters from EV-CDCs control samples. Statistical differences between Zq values of sample groups were evaluated by paired *t*-test. Significant protein abundance change was set at a value of *p* < 0.05. Plots show the integrated Zq values from the sample groups for those proteins significantly up- (red) or down-regulated (blue) in IFNγ/EV-CDCs vs. EV-CDCs. **(B)** Significantly changed proteins were classified and annotated by Reactome in the top level pathways. **(C)** A total of 952 proteins (number of peptides, Np ≥ 2 at 1% FDR) quantified by iTRAQ-based proteomics were subjected to PCA. Left: Score plot for PC1 (40% variance explained) vs. PC3 (16% variance explained). Right: Score plot for PC2 (32.2% variance explained) vs. PC5 (3.4% variance explained). Data display 95% confidence regions. Animal origin (*n* = 4) is indicated on the plots as p1–p4. IFNγ/EV-CDCs (treated) and EV-CDCs (untreated, control) samples are indicated in red and green, respectively.

Once the EV-CDC protein cargo was classified, we resorted to a multiplexed quantitative proteomic approach, which offers an extensive dynamic range and great proteome coverage, allowing the simultaneous identification and quantification of hundreds of proteins in the same experiment. This methodology offers an important advantage for the analysis of limited sample amounts (Edwards and Haas, 2016; Jylhä et al., 2018), as in EV-CDC case. In this analysis, protein abundance changes in the IFNγ/EV-CDCs were calculated in relation to the average values of each protein quantified in EV-CDCs (log2-ratio) and expressed in units of standard deviation (Zq). Our results showed that a total of 37 proteins were differentially expressed when EV-CDCs and IFNγ/EV-CDCs were compared (*p* ≤ 0.05). Among the significantly increased proteins in IFNγ/EV-CDCs, we identified SLA, PSMB9, Tubulin alpha chain, TUBA1B, CCT5, ASNS, CDH2, SEPT9, COC100625519, IL6, C14orf166, PCNA, SERPINA7, ACAT2, NCL, LIPG, NUDC, PSMA2, PLG, RAB1A, ACTR2, and BLVRB (*n* = 22). Conversely, among the significantly decreased proteins in IFNγ/EV-CDCs, we identified ALDOC, WDR1, COL14A1, CAPG, SEPT6, PFDN4, PSMA6, TPM3, SORBS1, ARCN1, PUF60, CHORDC1, LOC100519984, CASP3, and PSMB6 (*n* = 15) (Figure 3A).

In order to validate these proteomic results, ELISA tests were performed in EV-CDCs and IFNγ/EV-CDCs. Unfortunately, there are few available reagents for swine protein detection, and this study was limited to the validation of IL6 expression in EVs from three pigs by ELISA. According to proteomic analysis, ELISA tests showed an increase in IL6 in IFNγ/EV-CDCs samples: 9.52 × 10^–8^ ± 4.68 × 10^–8^ ng/particle in EV-CDCs (*n* = 3) and 1.37 × 10^–7^ ± 8.43 × 10^–8^ in IFNγ/EV-CDCs (*n* = 3). It is important to note that the number of samples did not allow a proper statistical analysis, and further validations are required.

The differentially expressed proteins were then classified according to top-level Reactome pathways (Figure 3B). Of note, proteins such as PSMB6, ACTR2, PSMA6, TUBA1B, IL6, PSMA2, CASP3, ALDOC, and PSMB9 were classified in the pathway *Immune System* where six of them were found to be increased (PSMB6, ACTR2, TUBA1B, IL6, PSMA2, and PSMB9) and three decreased (PSMA6, CASP3, and ALDOC) in IFNγ/EV-CDCs vs. control EV-CDCs.

Finally, the unsupervised evaluation of proteomic results through principal component analyses (PCA) showed considerable differences between EV-CDCs and IFNγ/EV-CDCs (Figure 3C). Additionally, PCA analyses revealed that the distribution of main protein components (PC1 vs. PC2) from the same animal under both treatments behaved similarly, highlighting a distinctive individual EV-CDC proteome background regardless of IFNγ priming (Figure 3C, left). Despite individual differences among animals, IFNγ-priming of CDCs caused an important effect on the EV proteome (Figure 3C, right).

These proteomic results prompted us to complete the characterization of EV-CDCs and IFNγ/EV-CDCs based on miRNA analysis.

### Real-Time Quantification of miRNAs on EV-CDCs and IFNγ/EV-CDCs

**FIGURE 4:**
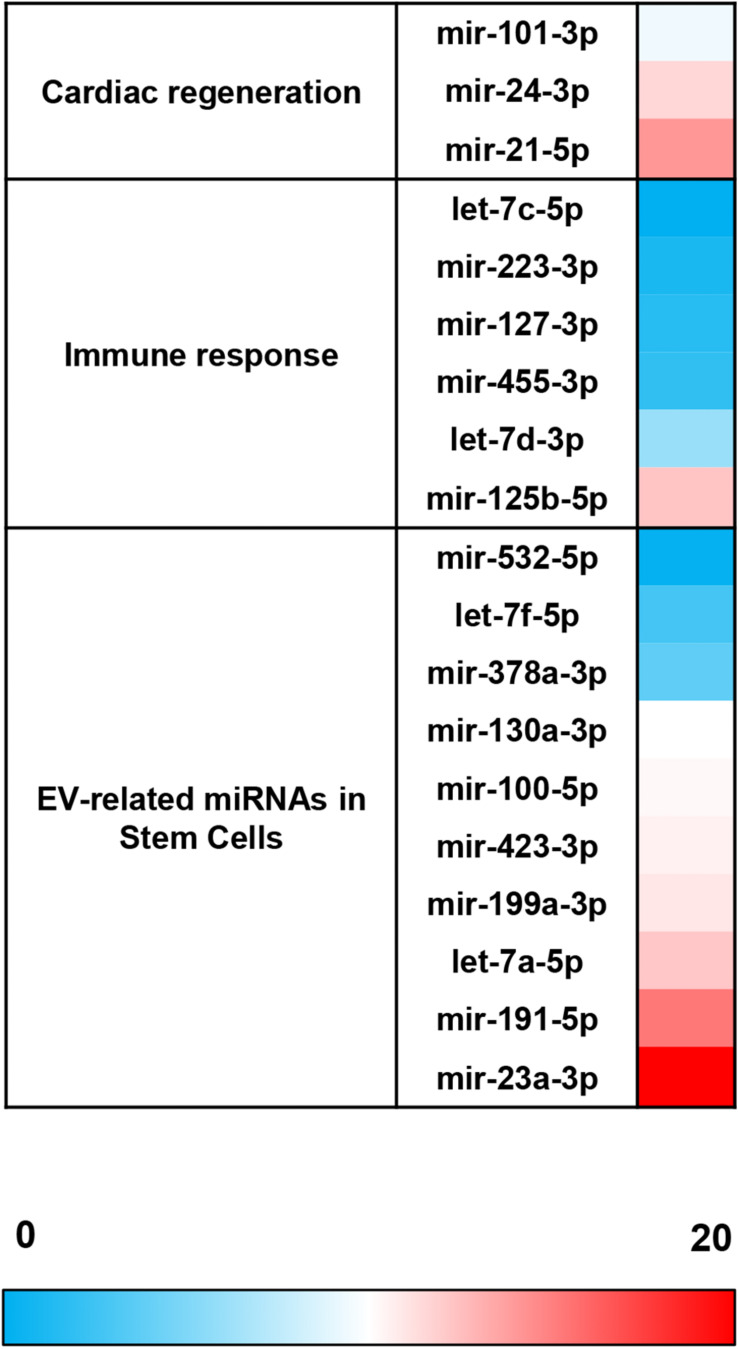
miRNA expression abundance in EV-CDCs. miRNA expression was analyzed by qPCR. Total RNA was isolated from EV-CDCs, and the amplification products were analyzed by the 2^–ΔCt^ method using the most stable miRNAs as endogenous controls. The statistical analysis was performed using a Thermo Fisher Cloud Analysis version 1.1. The figure shows the abundance of miRNAs in terms of 2^–ΔCt^, from less (blue) to more abundant (red), grouped by our selected miRNA panel.

The comparative analysis of miRNAs was performed in a selected panel of cardiac-related miRNAs, immune-related miRNAs, and miRNAs associated to EVs from adult/mesenchymal and stem/stromal cells. The evaluation of miRNAs by qPCR revealed that 25 of 44 total evaluated miRNAs were not expressed (or expressed below the detection limit) in EV-CDCs (Table 1). Interestingly, mir-23a-3p, mir-191-5p, mir-21-5p, mir-125b-5p, and let-7a-5p were abundantly expressed (2^–ΔCt^ > 4) in EV-CDCs (Figure 4).

**TABLE 1:** Panel of miRNA transcriptomic analysis of extracellular vesicles derived from CDCs (EV-CDCs).

	**miRNAs**		**References**
	**Expressed in EV-CDCs**	**Not expressed in EV-CDCs**	
**Cardiac-related miRNAs**	mir-101-3p mir-21-5p mir-24-3p	mir-133a-5p mir-15b-5p mir-208b-3p mir-29b-3p mir-29c-3p mir-34a-5p mir-34c-5p mir-92a-3p	Bernardo et al., 2015; Chistiakov et al., 2016; Zhu et al., 2016
**Immune-related miRNAs**	let-7c-5p let-7d-3p mir-125b-5p mir-127-3p mir-223-3p mir-455-3p	let-7d-3p mir-126-3p mir-126-5p mir-132-3p mir-137-3p mir-139-3p mir-142-5p mir-145-3p mir-150-5p mir-487b-5p	O’Neill et al., 2011; Marques-Rocha et al., 2015
**EV-MSCs-related miRNAs**	let-7a-5p let-7f-5p mir-100-5p mir-130a-3p mir-191-5p mir-199a-3p mir-23a-3p mir-378a-3p mir-423-3p mir-532-5p	let-7i-3p mir-146a-5p mir-148a-3p mir-148a-5p mir-29a-5p mir-424-5p mir-451a	Eirin et al., 2014; Fernández-Messina et al., 2015; Fafián-Labora et al., 2017; Zhao et al., 2017; Ferguson et al., 2018; Namazi et al., 2018a

*A deep bibliographic research was performed to select a list of miRNAs for transcriptomic analysis of EV-CDCs. These miRNAs were classified into different groups: cardiac-related miRNAs, immune-related miRNAs, and miRNAs associated with extracellular vesicles from adult/mesenchymal and stem/stromal cells. Real-time quantitative PCR (qPCR) was carried out to analyze miRNA expression. The table shows miRNA classification according to their expression in EV-CDCs.*

**FIGURE 5:**
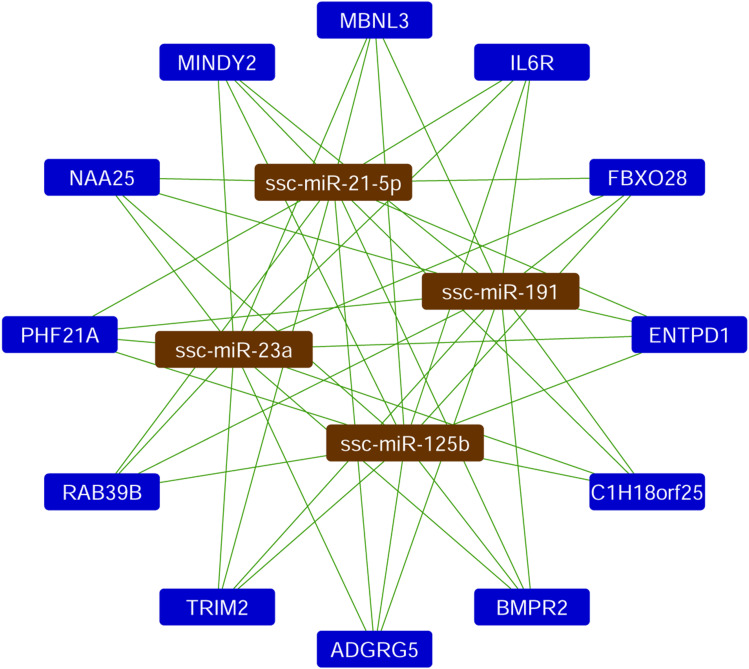
Analysis of miRNA target network. The miRNet analysis was performed in the four top-abundant expression analyzed miRNAs. The network shows the interactions with the *Sus scrofa* target genes.

Based on the quantification of miRNAs, the top four most abundant miRNAs in EV-CDCs (mir-23a-3p, mir-191-5p, mir-21-5p, and mir-125b-5p) were further analyzed using a *Sus scrofa* database with the miRNet tool. As shown in Figure 5, this analysis revealed that the following genes could be targeted by each of these top-abundant miRNAs: *IL6R*, *ADGRG5*, *C1H18orf25*, *NAA25*, *PHF21A*, *MINDY2*, *RAB39B*, *TRIM2*, *MBNL3*, *ENTPD1*, *FBXO28*, and *BMPR2.*

**FIGURE 6:**
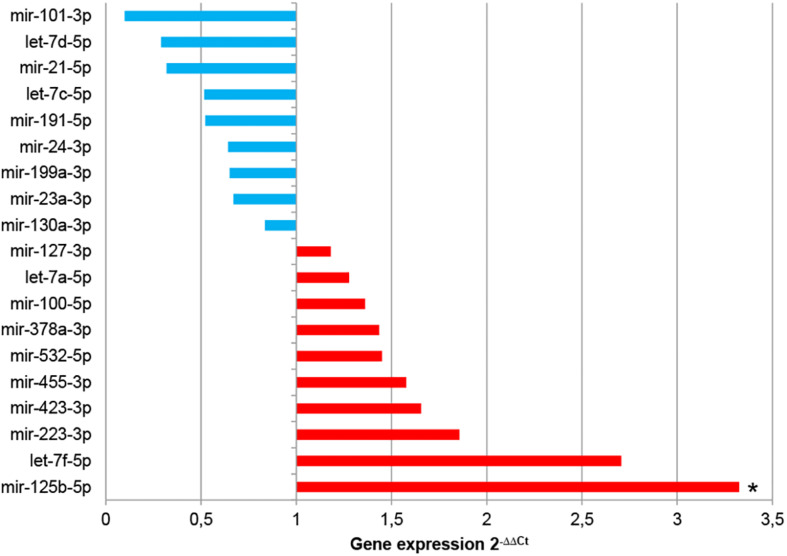
Comparative analysis of miRNA expression between EV-CDCs and IFNγ/EV-CDCs. The comparative analysis of miRNA expression was performed by qPCR. Total RNA was isolated from EV-CDCs and IFNγ/EV-CDCs, and amplification products were compared by the 2^–ΔΔCt^ method using the most stable miRNAs as endogenous controls. The statistical analysis was performed using a Thermo Fisher Cloud Analysis version 1.1. Graphs show 2^–ΔΔCt^. *p ≤ 0.05.

The analysis of miRNA expression levels was also used to compare EV-CDCs and IFNγ/EV-CDCs. With this aim, 2^–ΔΔCt^ calculation was performed using EV-CDCs as the “control group.” Paired *t*-test analysis demonstrated a significant increase in mir-125b-5p in IFNγ/EV-CDCs. Additionally, this analysis showed an increase (although non-significant) in IFNγ/EV-CDCs of let-7f-5p, mir-223-3p, mir-423-3p, mir-455-3p, mir-532-5p, mir-378a-3p, mir-100-5p, let-7a-5p, and mir-127-3p, together with a non-significant decrease in mir-130a-3p, mir-23a-3p, mir-199a-3p, mir-24-3p, mir-191-5p, let-7c-5p, mir-21-5p, and let-7d-5p, and mir-101-3p (Figure 6).

**FIGURE 7:**
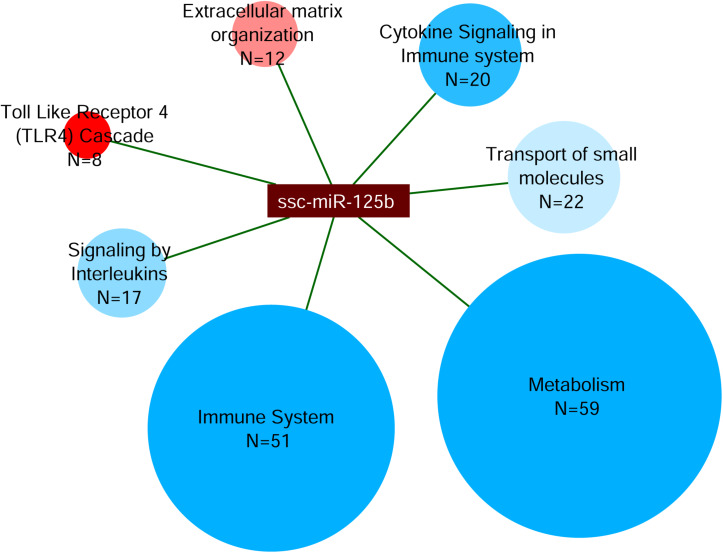
Analysis of miRNA target network of mir-125b. The miRNet analysis was performed on the up-regulated mir-125b in IFNγ/EV-CDCs vs. EV-CDCs. Targeted genes were classified by g:Profiler in Reactome pathways. The network shows the interactions between mir-125b and the Reactome terms (ellipses). Ellipsis size represents the number of proteins in each category (N), while their color represents the significance level of each category, from less (blue) to more statistically significant (red).

Finally, miRNet analysis was carried out for the differentially expressed mir-125b. This analysis revealed a total of 1,367 interactions with *Sus scrofa* target genes. The 510 genes with more than 150 *experiment scores* were further classified by the webserver g:Profiler in the Reactome pathways (Figure 7). This classification showed that 59 targeted genes were categorized in *Metabolism*; 51 targeted genes were categorized in *Immune System*; 22 targeted genes in *Transport of Small Molecules*; 20 targeted genes in *Cytokine Signaling in Immune System*; 17 targeted genes in *Signaling by Interleukins*, 12 targeted genes in *Extracellular Matrix Organization*; and 8 targeted genes in *Toll-Like Receptor 4 (TLR4) Cascade.*

### *In vitro* Effect of EV-CDCs and IFNγ/EV-CDCs in Lymphocyte Differentiation and Activation

**FIGURE 8:**
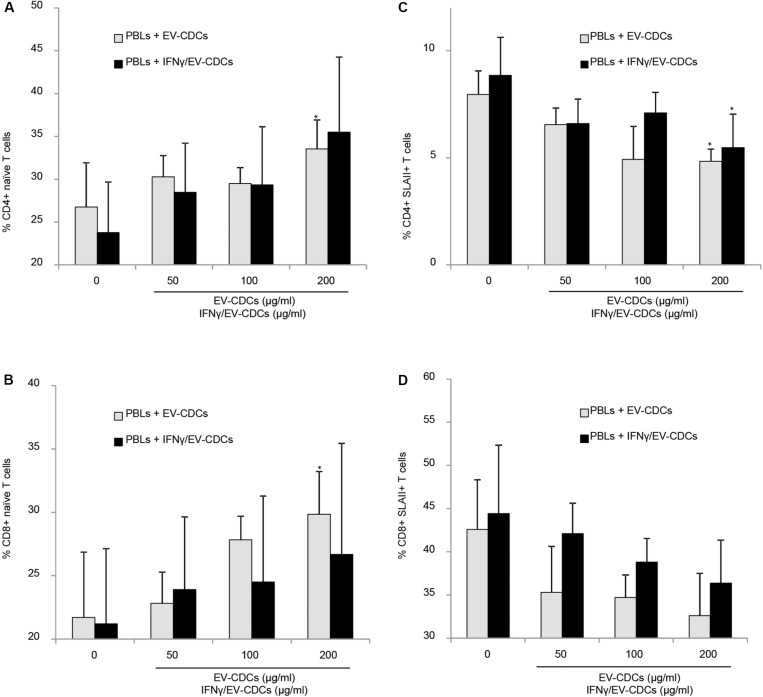
*In vitro* peripheral blood lymphocyte (PBL) activation and differentiation assays in co-culture with EV-CDCs and IFNγ/EV-CDCs. Peripheral blood lymphocytes (PBLs) were isolated from blood samples by density gradient and co-cultured with 0 (control), 50, 100, and 200 μg/ml of EV-CDC (gray bars) and IFNγ/EV-CDC (black bars) protein for 3 days. Lymphocyte activation/differentiation was analyzed by flow cytometry on CD4+ **(A,C)** and CD8+ T-cell subpopulations **(B,D)**, using naïve T-cell markers (CD45RA+, CD27+) and activation marker (SLAII). Paired *t*-test was used to compare doses of EVs to negative controls (*p < 0.05).

The flow cytometry analysis of PBLs co-cultured with EV-CDCs and IFNγ/EV-CDCs was performed on day 3. Activation/differentiation markers were analyzed on CD4+ and CD8+ T-cell subsets. The first analysis was focused on CD45RA and CD27 expression. The percentages of naïve CD8+ T cells (CD45RA+/CD27+) and naïve CD4+ T cells (CD45RA+) were compared in a paired *t*-test using PBLs without EVs as negative controls. Our results demonstrated that EV-CDCs at 200 μg/ml counteracted the *in vitro* differentiation of CD4+ T cells and CD8+ T cells toward effector/memory cells. The percentages of naïve CD4+ T cells and CD8+ T cells were significantly higher than the negative controls (PBLs without EVs) (Figures 8A,B, respectively). Similarly, IFNγ/EV-CDCs partially counteracted the *in vitro* differentiation of CD4+ T cells and CD8+ T cells. However, the differences of percentage of naïve T cells between EV-CDCs and IFNγ/EV-CDCs were not statistically significant (Figures 8A,B).

The second analysis was focused on Swine Leukocyte Antigen class II (SLAII) expressed in CD4+ and CD8+ T-cell subsets. The surface expression of MHC class II can be defined as an activation marker on T-cell subsets (Revenfeld et al., 2017). In this study, we quantified the percentages of CD4+ SLAII+ T cells and CD8+ SLAII+ T cells on PBLs co-cultured with EVs. Our results showed a significant decrease in activated CD4+ T cells in PBLs co-cultured with EV-CDCs and IFNγ/EV-CDCs at 200 μg/ml (Figure 8C). Additionally, PBLs co-cultured with different EVs showed a decrease in CD8+ SLAII+ T cells (Figure 8D), although no significant difference was found when EV-CDCs and IFNγ/EV-CDCs were compared.

## Discussion

Large animal models in preclinical research are essential for a successful clinical translation of advanced therapies. Porcine models have been widely used in cardiovascular research to evaluate different administration routes (McCall et al., 2012), stem cell-based therapies (Bolli et al., 2013; Ye et al., 2014; Crisostomo et al., 2015), and to identify biomarkers under controlled experimental conditions (Koudstaal et al., 2014; Blázquez et al., 2018; López et al., 2019). Obviously, the translation of preclinical results to clinical trials is an arduous and challenging process. First, animal models cannot fully represent human disease, where risk factors and comorbidities play important roles. Second, therapeutic products (such as stem cells) are usually different in animal studies and clinical trials. For this reason, an exhaustive analysis of animal-derived therapeutic products is necessary prior to be used in preclinical models.

Nowadays, human-derived EVs from adult stem cells are very well studied; however, animal-derived EVs are poorly characterized, and this characterization is mandatory for a successful translation from animal models to humans. Based on that, the first goal of this study was to perform a deep proteomic and genomic analysis of porcine-derived EV-CDCs. Additionally, we hypothesized that priming *in vitro* cultured CDCs with inflammatory stimuli (such as IFNγ) may increase the therapeutic potential (immunomodulatory and/or pro-regenerative) of released vesicles. This hypothesis is based on previous studies in which primed MSCs with hypoxia, serum deprivation, or inflammatory cytokines produced soluble factors with immunomodulatory and pro-angiogenic properties (Oh et al., 2008; DelaRosa et al., 2009; Di Trapani et al., 2016; Song et al., 2017; Ragni et al., 2019; Showalter et al., 2019).

Our first set of results has demonstrated that *in vitro* culture conditions for CDCs and vesicle isolations were optimal and provided a satisfactory enrichment of EV-CDCs. The nano flow-cytometry analyses led us to demonstrate that our EV-CDC preparations were enriched in small EVs (ranging from 40 to 200 nm). Of note, high-sensitivity nano-flow cytometry has recently demonstrated to be comparable to electron microscopy, notably reducing costs, sample preparation time, and increasing statistical power of analysis (Tian et al., 2018). In our case, although the isolation protocols demonstrated significant enrichment of *extracellular exosome* proteins, this methodology did not exclude the co-purification of extracellular matrix proteins, such as collagens, and other proteins, such as Vinculin, Filamin A, or Fibronectin 1.

The enrichment analysis of top-abundant protein (Np ≥ 20) in EV-CDCs by Reactome revealed an over-representation in three top-level categories: *Immune System*, *Homeostasis*, and *Muscle Contraction.* This enrichment analysis highlights the hypothetical involvement of proteins and clusters of proteins in the therapeutic effect of these vesicles. For example, HSP90 (classified in *Immune System*) has been found to be involved in the modulation of cardiac ventricular hypertrophy (Tamura et al., 2019), and SPARC (classified in *Hemostasis*) has been found to be involved in the improvement of cardiac function after myocardial infarction (Deckx et al., 2019).

This study was also focused on the comparative analysis between EVs released from *in vitro* cultured CDCs and EVs from IFNγ-primed CDCs. NanoFCM results did not show significant differences between IFNγ-primed and control EV-CDCs in terms of size profile, nor particle concentration (Figure 1). The idea of inflammatory priming to increase the immunomodulatory effect of cells has been first described to generate anti-inflammatory cells and, more recently, to generate anti-inflammatory vesicles (Di Trapani et al., 2016; Song et al., 2017; Showalter et al., 2019). Our matched-paired comparative analysis revealed significant differences in 37 proteins and, although many of these proteins may deserve a proper discussion, we focused our interest in some of the proteins categorized in the *Immune System* pathway by Reactome.

Our results showed differential expressions in four different proteasome subunits: PSMA2, PSMB9 (increased), PSMB6, and PSMA6 (decreased). It is well known that IFNγ and other pro-inflammatory signals are involved in the formation of immunoproteasome, which is derived from the constitutive proteasome (Strehl et al., 2005). Moreover, the presence of co-purified proteasome subunits has been described in exosomes derived from MSCs, and the authors suggested that these proteasome subunits “*could synergize with other constituents to ameliorate tissue damage*” (Lai et al., 2012).

Apart from proteasome subunits, the comparative analysis showed a significant increase in ACTR2 (actin-related protein 2) in IFNγ/EV-CDCs. This protein is also classified in the *Immune System* pathway (*R-SSC-168256*) and is a key component of the Arp2/3 complex, which is involved in actin polymerization (Suetsugu and Takenawa, 2003). According to Exocarta (Mathivanan and Simpson, 2009), this protein was previously described in very different tissues and cell types. Moreover, Reactome pathway analysis demonstrated that this protein is functionally classified in very different pathways, such as *EPHB-Mediated Forward Signaling*, *Regulation of Actin Dynamics for Phagocytic Cup Formation*, and *RHO GTPases Activate WASPs and WAVEs* (Mathivanan and Simpson, 2009). Unfortunately, it is difficult to elucidate the consequences of this change, and the functional relevance of this protein requires further investigations.

Among the significantly increased proteins in IFNγ/EV-CDCs classified in the *Immune System* pathway, the increase in IL6 is especially relevant. The biological role of this cytokine has always been contradictory. On the one hand, IL6 has been considered as a pro-inflammatory cytokine, participating in the development of coronary heart disease, obesity or diabetes (Fuster and Walsh, 2014). In contrast, it has been associated with the alternative activation of macrophages (Mauer et al., 2014) and with an atheroprotective effect against inflammatory vascular disorders (Elhage et al., 2001; Schieffer et al., 2004). Additionally, it was defined as a “myokine” with anti-inflammatory effects during exercise (Pedersen, 2006). In the context of inflammatory-primed MSCs, IL6 secretion has been considered as an anti-inflammatory molecule (Xing et al., 1998). So, here, we hypothesize that the presence of IL6 in EV-CDCs, as well as the increase in IFNγ/EV-CDCs, may have a therapeutic effect in the control of acute inflammatory responses in myocardial infarction. However, we should also keep in mind that many different studies have experimentally demonstrated that IL6R signaling has an adverse effect and a key role in the development of stroke (IL6R Genetics Consortium Emerging Risk Factors Collaboration et al., 2012; Schuett et al., 2012).

In short, the proteomic analysis of EV-CDCs and IFNγ/EV-CDCs showed a myriad of different proteins with different functions. The bioinformatic and biostatistical analyses in top-abundant proteins (Np ≥ 20) suggest the contribution of clusters of proteins in immune-related and cardiac-related process. Once the proteomic profile of vesicles was identified, and considering that the therapeutic effect of exosomes from CDCs is also mediated by miRNAs (Namazi et al., 2018b), here, we performed a quantitative and comparative analysis in a panel of miRNAs. This panel was selected for their association with cardiac regeneration, immune response, and expression in EVs.

In this analysis, mir-23a-3p, mir-191-5p, mir-21-5p, mir-125b-5p, and let-7a-5p were identified as the top-abundant miRNAs in EV-CDCs. Although further experimental validations are needed to assess the impact of these miRNAs, the *in silico* analysis by miRNet revealed that *IL6R* is a target gene of the four top-abundant miRNAs.

The comparative analysis of miRNAs in EV-CDCs and IFNγ/EV-CDCs showed a statistically significant difference in the expression of mir-125b-5p. Previous studies demonstrated that the presence of this miRNA is positively correlated with circulating inflammatory cytokines in patients with chronic obstructive pulmonary disease (Hu et al., 2017). This may explain, at least in part, the increased release of mir-125b-5p under IFNγ stimuli. Additionally, mir-125b has been described as a cardioprotective miRNA that participates in cardiac regeneration after myocardial infarction (Wang et al., 2014), having a key role in the regulation of cardiomyocyte survival during acute myocardial infarction (Bayoumi et al., 2018). So, the local administration of IFNγ-primed vesicles may have increased therapeutic potential in infarcted patients.

The target network for mir-125b-5p was finally analyzed by miRNet. This analysis identified 1,367 miRNA–gene target interactions. Although this *in silico* study needs to be corroborated by functional studies, the 510 genes with the highest *experiment score* (>150) were categorized by Reactome. Most of the targeted genes were classified in the terms of *Metabolism* and *Immune System*.

Our *in silico* analysis also suggested the hypothetical involvement of the four top-abundant miRNAs in EV-CDCs, including mir-125b-5p (differentially expressed between EV-CDCs and IFNγ/EV-CDCs), in the regulation of the *IL6R* gene. According to the Interleukin-6 Receptor Mendelian Randomisation Consortium, *IL6R* has been considered a target for coronary heart disease: “*IL6R blockade could provide a novel therapeutic approach to prevention of coronary heart disease.*” Nowadays, monoclonal antibodies against IL6R, such as tocilizumab, have been considered as a therapeutic strategy for prevention of coronary heart disease (Carroll, 2018). Based on that, the optimization of *in vitro* culture conditions for EV-CDC isolation may have a therapeutic relevance for targeting IL6R and subsequently in inflammatory-mediated diseases. Moreover, it would be interesting to analyze additional miRNA targeting *IL6R* in EV-CDCs (i.e., mir-34b-3p, mir-124-3p).

Our proteomic and genomic analysis was finally completed with an *in vitro* study to determine the immunomodulatory effect of EV-CDCs and IFNγ/EV-CDCs on lymphocyte subsets. Owing to the limited availability of reagents for this animal model, this study was focused on CD4+ and CD8+ T-cell subsets. The *in vitro* differentiation and activation markers were analyzed in PBLs co-cultured with EV-CDCs and IFNγ/EV-CDCs. Our results demonstrated that EV-CDCs counteracted the *in vitro* differentiation of CD4+ and CD8+ T-cells toward an effector memory phenotype and reduced the expression of activation markers. This result agrees with our previous studies using EVs derived from endometrial stem cells (Álvarez et al., 2018; Marinaro et al., 2019). In fact, similar to Álvarez et al. (2018) here, we could also assert that EV-CDCs have an “*inhibitory effect against CD4+ T cell activation.*” It is important to note that, under these experimental conditions, paired *t*-test did not reveal significant differences between control EV-CDCs and IFNγ/EV-CDCs.

In summary, here, we demonstrate that *in vitro* cultured CDCs release vesicles that are enriched in immune-related proteins. On the one hand, the content of these EV-CDCs can modify *in vitro* the immunomodulatory status of PBLs. Within the protein content, the abundance and the increased expression of IL6 in IFNγ/EV-CDCs are especially relevant. According to preclinical models, which demonstrated an IL6-dependent M2b polarization using IFNγ pre-conditioned MSCs (Philipp et al., 2018), here, we hypothesize that IL6 expression in EV-CDCs may have a key role in the regulation of macrophage and/or neutrophil polarization. On the other hand, miRNA analyses pinpoint the abundance and the differential expression of mir-125b-5p, which target genes involved in the *Immune System* process, including *IL6R*. Altogether, the proteomic and genomic results point out the hypothetical involvement of these vesicles in the regulation of IL6/IL6R axis and, subsequently, in inflammatory-mediated diseases.

## Data Availability Statement

The mass spectrometry proteomics data can be found in ProteomeXchange (http://www.proteomexchange.org/) with identifier PXD016434.

## Ethics Statement

The animal study was reviewed and approved by the Animal Welfare and Ethics Committee of the Jesús Usón Minimally Invasive Surgery Centre, in accordance with the recommendations outlined by the local government (Junta de Extremadura), and the EU Directive 2010/63/EU of the European Parliament on the protection of animals used for scientific purposes.

## Author Contributions

EL, FM, MP, MG-S, FS-M, IJ, and JC conceived and designed the experiments. EL, FM, MP, MG-S, IJ, JV, VÁ, VC, LF-P, VP, EP, and JC performed the experiments and analyzed the data. EL, FM, MG-S, IJ, and JC wrote the manuscript.

## Conflict of Interest

The authors declare that the research was conducted in the absence of any commercial or financial relationships that could be construed as a potential conflict of interest.

## Footnotes

1 https://david.ncifcrf.gov/home.jsp

2 https://reactome.org/

3 https://www.metaboanalyst.ca/

4 https://www.mirnet.ca/miRNet/home.xhtml

5 http://david.abcc.ncifcrf.gov

6 http://www.exocarta.org/

## Abbreviations

CDCs: cardiosphere-derived cells
DMEM: Dulbecco’s modified Eagle’s medium
EV-CDCs: extracellular vesicles from cardiosphere-derived cells
EVs: extracellular vesicles
FDR: false discovery rate
IFN γ: interferon gamma
IFN γ /EV-end MSCs: extracellular vesicles from IFN γ-primed cardiosphere-derived cells
IPA: ingenuity pathway analysis
iTRAQ: isobaric Tags for Relative and Absolute Quantitation
LC-MS/MS: liquid chromatography-tandem mass spectrometry
MSCs: mesenchymal stem cells
Np: number of peptides
PBS: phosphate-buffered saline
PCA: principal component analysis
SBT: Systems Biology Triangle
WSPP, weighted spectrum: peptide protein.
