# Supplementary material for: The Immunomodulatory Signature of Extracellular Vesicles From Cardiosphere-Derived Cells: A Proteomic and miRNA Profiling
Source: Front Cell Dev Biol. 2020 Jun 9;8:321. doi: 10.3389/fcell.2020.00321 (PMC7295954; doi:10.3389/fcell.2020.00321)
Supplement: Supplementary file 1 [file Data_Sheet_1.DOCX]

Supplementary Material

Supplementary tables

| **Category** | **Protein** | **Peptide count** | **Protein** | **Peptide count** | **Category** | **Protein** | **Peptide count** | **Protein** | **Peptide count** |
| --- | --- | --- | --- | --- | --- | --- | --- | --- | --- |
| 1- Transmembrane  or GPI-anchored proteins associated  to plasma  membrane and/or endosomes | CD63 | 5 | ITGA8 | 6 | 4- Transmembrane,  lipid bound  and soluble proteins associated to other intracellular compartments than PM/endosomes | HIST1H2AH | 2 | HSP90B1 | 18 |
|  | CD81 | 2 | ITGB1 | 13 |  | HIST1H2BA | 1 | HSPA5 | 21 |
|  | CD151 | 1 | LAMP2 | 3 |  | HIST1H2BH | 6 | ACTN1 | 42 |
|  | CD9 | 1 | SDC1 | 1 |  | HIST1H3D | 1 | ACTN2 | 12 |
|  | CD90 | 3 | SDC2 | 1 |  | HIST1H4A | 8 | ACTN3 | 2 |
|  | GNA13 | 1 | SDC4 | 3 |  | LMNA | 50 | ACTN4 | 60 |
|  | GNAI2 | 6 | NT5E | 2 |  | CANX | 2 |  |  |
|  |  |  |  |  |  |  |  |  |  |
| 2- Cytosolic  proteins recovered in EVs | PDCD6IP | 11 | SDCBP | 5 | 5- Secreted proteins recovered with EVs | TGFB2 | 1 | COL3A1 | 62 |
|  | CAV1 | 1 | HSPA1A | 10 |  | TGFBI | 11 | COL4A1 | 6 |
|  | EHD1 | 3 | ACTA1 | 4 |  | PDGFA | 1 | COL4A2 | 10 |
|  | EHD2 | 7 | ACTA2 | 34 |  | IL6 | 2 | COL4A4 | 1 |
|  | RHOA | 4 | ACTC1 | 2 |  | FN1 | 77 | COL5A1 | 27 |
|  | ANXA1 | 11 | ACTG1 | 18 |  | COL11A1 | 1 | COL5A2 | 34 |
|  | ANXA11 | 3 | TUBA1A | 1 |  | COL11A2 | 2 | COL5A3 | 3 |
|  | ANXA2 | 19 | TUBA1B | 18 |  | COL12A1 | 25 | COL6A1 | 19 |
|  | ANXA4 | 2 | TUBA1C | 1 |  | COL14A1 | 20 | COL6A2 | 26 |
|  | ANXA5 | 11 | TUBA4B | 1 |  | COL15A1 | 9 | COL6A3 | 31 |
|  | ANXA6 | 14 | TUBA8 | 1 |  | COL16A1 | 1 | COL7A1 | 1 |
|  | ANXA7 | 2 | GAPDH | 19 |  | COL18A1 | 2 | COL9A1 | 1 |
|  | HSP90AB1 | 17 |  |  |  | COL1A1 | 64 | MFGE8 | 12 |
| 3- Major components of non-EV co-isolated structures | APOA1 | 7 | APOB | 5 |  | COL1A2 | 103 | LGALS3BP | 7 |
|  | APOA2 | 1 | ALB | 29 |  | COL2A1 | 7 | AHSG | 7 |

**Supplementary table 1. EV markers according to MISEV2018 guidelines.** Proteomic analysis identified the protein cargo of EV-CDCs and the identified peptides were counted and classified according to the categories established by MISEV2018 guidelines.

| **miRNA** | **Assay ID/Name** | **miRNA** | **Assay ID/Name** |
| --- | --- | --- | --- |
| **let-7a-5p** | [478575_mir](https://www.thermofisher.com/order/genome-database/details/microrna/478575_mir?CID=&ICID=&subtype=) | **miR-15b** | [478313_mir](https://www.thermofisher.com/order/genome-database/details/microrna/478313_mir?CID=&ICID=&subtype=) |
| **let-7c** | [478577_mir](https://www.thermofisher.com/order/genome-database/details/microrna/478577_mir?CID=&ICID=&subtype=) | **miR-191-5p** | [477952_mir](https://www.thermofisher.com/order/genome-database/details/microrna/477952_mir?CID=&ICID=&subtype=) |
| **let-7d-3p** | [477848_mir](https://www.thermofisher.com/order/genome-database/details/microrna/477848_mir?CID=&ICID=&subtype=) | **miR-199a-3p** | [477961_mir](https://www.thermofisher.com/order/genome-database/details/microrna/477961_mir?CID=&ICID=&subtype=) |
| **let-7d-5p** | [478439_mir](https://www.thermofisher.com/order/genome-database/details/microrna/478439_mir?CID=&ICID=&subtype=) | **miR-208b** | [477806_mir](https://www.thermofisher.com/order/genome-database/details/microrna/477806_mir?CID=&ICID=&subtype=) |
| **let-7f** | [478578_mir](https://www.thermofisher.com/order/genome-database/details/microrna/478578_mir?CID=&ICID=&subtype=) | **miR-21-5p** | [477975_mir](https://www.thermofisher.com/order/genome-database/details/microrna/477975_mir?CID=&ICID=&subtype=) |
| **let-7i-3p** | [477862_mir](https://www.thermofisher.com/order/genome-database/details/microrna/477862_mir?CID=&ICID=&subtype=) | **miR-223** | [rno481007_mir](https://www.thermofisher.com/order/genome-database/details/microrna/rno481007_mir?CID=&ICID=&subtype=) |
| **miR-100-5p** | [478224_mir](https://www.thermofisher.com/order/genome-database/details/microrna/478224_mir?CID=&ICID=&subtype=) | **miR-22-3p** | [477985_mir](https://www.thermofisher.com/order/genome-database/details/microrna/477985_mir?CID=&ICID=&subtype=) |
| **miR-101** | [477863_mir](https://www.thermofisher.com/order/genome-database/details/microrna/477863_mir?CID=&ICID=&subtype=) | **miR-23a-3p** | [478532_mir](https://www.thermofisher.com/order/genome-database/details/microrna/478532_mir?CID=&ICID=&subtype=) |
| **miR-125b-5p** | [477885_mir](https://www.thermofisher.com/order/genome-database/details/microrna/477885_mir?CID=&ICID=&subtype=) | **miR-24** | [477992_mir](https://www.thermofisher.com/order/genome-database/details/microrna/477992_mir?CID=&ICID=&subtype=) |
| **miR-126-3p** | [477887_mir](https://www.thermofisher.com/order/genome-database/details/microrna/477887_mir?CID=&ICID=&subtype=) | **miR-29a-5p** | [478002_mir](https://www.thermofisher.com/order/genome-database/details/microrna/478002_mir?CID=&ICID=&subtype=) |
| **miR-126-5p** | [477888_mir](https://www.thermofisher.com/order/genome-database/details/microrna/477888_mir?CID=&ICID=&subtype=) | **miR-29b-3p** | [478369_mir](https://www.thermofisher.com/order/genome-database/details/microrna/478369_mir?CID=&ICID=&subtype=) |
| **miR-127-3p** | [477889_mir](https://www.thermofisher.com/order/genome-database/details/microrna/477889_mir?CID=&ICID=&subtype=) | **miR-29c-3p** | [479229_mir](https://www.thermofisher.com/order/genome-database/details/microrna/479229_mir?CID=&ICID=&subtype=) |
| **miR-130a-3p** | [477851_mir](https://www.thermofisher.com/order/genome-database/details/microrna/477851_mir?CID=&ICID=&subtype=) | **miR-34a** | [478048_mir](https://www.thermofisher.com/order/genome-database/details/microrna/478048_mir?CID=&ICID=&subtype=) |
| **miR-132** | [477900_mir](https://www.thermofisher.com/order/genome-database/details/microrna/477900_mir?CID=&ICID=&subtype=) | **miR-34c** | [478052_mir](https://www.thermofisher.com/order/genome-database/details/microrna/478052_mir?CID=&ICID=&subtype=) |
| **miR-133a** | [478706_mir](https://www.thermofisher.com/order/genome-database/details/microrna/478706_mir?CID=&ICID=&subtype=) | **miR-378** | [478349_mir](https://www.thermofisher.com/order/genome-database/details/microrna/478349_mir?CID=&ICID=&subtype=) |
| **miR-137** | [477904_mir](https://www.thermofisher.com/order/genome-database/details/microrna/477904_mir?CID=&ICID=&subtype=) | **miR-424-5p** | [478092_mir](https://www.thermofisher.com/order/genome-database/details/microrna/478092_mir?CID=&ICID=&subtype=) |
| **miR-139-3p** | [477906_mir](https://www.thermofisher.com/order/genome-database/details/microrna/477906_mir?CID=&ICID=&subtype=) | **miR-451a** | [478107_mir](https://www.thermofisher.com/order/genome-database/details/microrna/478107_mir?CID=&ICID=&subtype=) |
| **miR-142-5p** | [477911_mir](https://www.thermofisher.com/order/genome-database/details/microrna/477911_mir?CID=&ICID=&subtype=) | **miR-455-3p** | [478112_mir](https://www.thermofisher.com/order/genome-database/details/microrna/478112_mir?CID=&ICID=&subtype=) |
| **miR-145-5p** | [477915_mir](https://www.thermofisher.com/order/genome-database/details/microrna/477915_mir?CID=&ICID=&subtype=) | **miR-487b** | [478938_mir](https://www.thermofisher.com/order/genome-database/details/microrna/478938_mir?CID=&ICID=&subtype=) |
| **miR-146a** | [478399_mir](https://www.thermofisher.com/order/genome-database/details/microrna/478399_mir?CID=&ICID=&subtype=) | **miR-532-5p** | [478151_mir](https://www.thermofisher.com/order/genome-database/details/microrna/478151_mir?CID=&ICID=&subtype=) |
| **miR-148a-3p** | [477814_mir](https://www.thermofisher.com/order/genome-database/details/microrna/477814_mir?CID=&ICID=&subtype=) | **miR-92a** | [477827_mir](https://www.thermofisher.com/order/genome-database/details/microrna/477827_mir?CID=&ICID=&subtype=) |
| **miR-148a-5p** | [478718_mir](https://www.thermofisher.com/order/genome-database/details/microrna/478718_mir?CID=&ICID=&subtype=) | **miR-16** | [477860_mir](https://www.thermofisher.com/order/genome-database/details/microrna/477860_mir?CID=&ICID=&subtype=) |
| **miR-150** | [477918_mir](https://www.thermofisher.com/order/genome-database/details/microrna/477918_mir?CID=&ICID=&subtype=) | **miR-423** | [478327_mir](https://www.thermofisher.com/order/genome-database/details/microrna/478327_mir?CID=&ICID=&subtype=) |

**Supplementary table 2. Assays ID of commercial TaqMan miRNA Assays.**

**Supplementary table 3. Enrichment analysis of EV-CDCs identified proteins (n=932) using DAVID software**. Different annotation databases as Gene Ontology (GO), KEEG, REACTOME or BIOCARTA were used. “Count” means the number of proteins within the corresponding category annotation. The percentage represents the proportion of gene-coded proteins annotated within the category. The magnitude of the category enrichment is measured by the corresponding fold enrichment, and p values indicate the significance level. Only terms with p < 0.05 are shown. Benjamini-Hochberg False Discovery Rate (FDR) was used for multiple test correction. DAVID software is available at: <https://david.ncifcrf.gov/>. Supplementary Table 3 has been uploaded and is available as Data Sheet (excel files).
